# Experience with Cervical Cancer Screening and Views on HPV Self-Sampling Among Somali American Women

**DOI:** 10.1007/s11606-025-10080-0

**Published:** 2025-12-18

**Authors:** Rebekah Pratt, Christina Bliss Barsness, John Lin, Fatumastar Adan, Sumeiya Abdi, Sharif Mohamed, Huda Farah, Amanda Trofholz, Jay Desai, Kristi Fordyce, Rahel Ghebre, Anisa Ibrahim, Tim Ramer, Adam Szpiro, Bryan J. Weiner, Sophia Yohe, Rachel L. Winer

**Affiliations:** 1 University of Minnesota, Minneapolis, USA; 2 University of Washington, Seattle, USA; 3 Islamic Civic Society of America, Minneapolis, USA; 4 Minnesota Department of Health, Saint Paul, USA

**Keywords:** HPV self-testing, cervical cancer, screening, minoritized populations, qualitative, primary care

## Abstract

**Background:**

Somali American women have lower cervical cancer screening rates than the general US population. Human papillomavirus (HPV) testing on self-collected samples (HPV self-sampling) has the potential to address cervical cancer screening disparities affecting Somali American women. This study aimed to understand Somali American women’s perspectives on cervical cancer screening and HPV self-sampling.

**Methods:**

Forty-four Somali American women participated in six focus groups. Participants were between 30 and 65 years old and were eligible for cervical cancer screening. The discussions focused on women’s experiences with cervical cancer screening, barriers to screening, views on HPV self-sampling, and recommendations to increase screening participation.

**Findings:**

While some participants’ prior experiences with cervical cancer screening were positive, many reported coercive, distressing, and frightening experiences with screening. A range of barriers was reported, and these included fear, distrust, low awareness of cervical cancer, modesty concerns, being circumcised, and limited access, including not being offered screening. Participants viewed HPV self-sampling favorably, with minor concerns about test validity or ability to collect samples correctly. Overall, participants felt HPV self-sampling should be routinely offered to all Somali American patients.

**Conclusion:**

Offering HPV self-sampling to Somali American women could be an important tool to address barriers related to modesty and access to care and may be most effective implemented alongside education to raise awareness about cervical cancer. This modality may be particularly important for patients who have had traumatic or coercive screening experiences and for patients who have experienced female genital circumcision.

**Clinical Trial Registration:**

NCT05453006

## INTRODUCTION

Cervical cancer is the fourth most common cancer in women globally.^1^ In the USA, despite widespread availability of screening, approximately 13,000 cervical cancers are diagnosed annually,^2^ and more than half are in women who are not up-to-date with guideline-recommended screening intervals.^3–5^ Current US guidelines endorse human papillomavirus (HPV)-only testing as a preferred or recommended alternative to Pap-only or Pap/HPV testing.^6,7^ HPV-only testing is more sensitive than Pap testing for detecting cervical precancer and can be performed on either clinician-collected or self-collected samples with comparable accuracy.^8–10^ HPV self-sampling is highly acceptable to women and offers advantages of privacy and autonomy, the opportunity to be more involved in one’s own health care, and removing the discomfort of a pelvic examination.^11,12^ HPV self-sampling is endorsed by the World Health Organization (WHO) as a screening modality for cervical cancer^13^ and is a standard care option in multiple countries.^14^ HPV self-sampling was approved by the US Food and Drug Administration on May 15, 2024, for use in healthcare settings.

As a promising strategy for women who may otherwise opt out of screening,^15^ HPV self-sampling has the potential to address cervical cancer screening disparities. While over three-fourths of the screen-eligible US female population is current with cervical cancer screening, only one-fourth to one-half of Somali American women in the USA have up-to-date screening,^16–18^ with recent immigrants and older women having the lowest screening rates.^19,20^ There are approximately 85,000 Somali Americans living in Minnesota, USA, and research shows that a complex set of barriers contributes to low cervical cancer screening rates among Somali American women. These include a lack of provider skill or confidence to provide care to Somali women who have been circumcised,^21–24^ as well as multiple patient-level factors: limited health literacy,^25^ lack of knowledge about cancer generally,^26,27^ limited awareness about HPV and cervical cancer,^26–30^ cultural values and interpretations of religion,^25,31–34^ such as views on predestination and modesty concerns,^35^ and access to care, including a mistrust of healthcare providers and systems.^25,29,36^

HPV self-sampling addresses some barriers faced by Somali American women (e.g., modesty, concerns about circumcision) and has the potential to increase cervical cancer screenings and reduce cervical cancer rates in Somali communities. One previous study using mailed HPV self-sampling kits found that Somali American women who offered a kit were 14 times more likely to participate in cervical cancer screening than those who were offered usual care.^37^ Integrating HPV self-sampling into primary care visits could offer additional advantages beyond mail-in kits.^9,38^ For example, providers would be able to offer information and address misconceptions about HPV and cervical cancer^39^ and build a relationship with patients, which may increase patients’ willingness to receive follow-up care for abnormal HPV self-sampling test results.^40^ Interviews with providers at two Minneapolis-based clinics found that providers felt positively about clinic-based HPV self-sampling and felt all patients—including Somali American patients—would react favorably as well. Providers also had ideas about the implementation of HPV self-sampling in a clinic setting, including how to tailor the offering in a culturally appropriate way.^38^ However, the voices of Somali American patients also need to be centered in order to best integrate HPV self-sampling into primary care and to ensure that the increasing availability of HPV self-sampling does not lead to increased disparities. The current study provides results from focus groups conducted with Somali American patients about their experiences with cervical cancer screenings, barriers to screening and recommendations to increase screenings, and their views on how HPV self-sampling could potentially address cervical cancer screening disparities.

## METHODS

Focus groups were conducted through the Isbaar project, an ongoing clinical trial designed to assess the effectiveness and implementation of a clinic-based HPV self-sampling program to increase cervical cancer screening rates in Somali American women.^38^ Stages of the Isbaar project include (1) gathering qualitative interviews from primary care providers^38^ and focus groups with Somali American women to understand barriers and facilitators to promoting HPV self-sampling; (2) implementing HPV self-sampling alongside usual care in intervention clinics; and (3) assessing the implementation of HPV self-sampling to inform future implementation in other primary care settings.

### Participants

Between June and August 2022, the study conducted 6 focus groups of Somali American women (*n* = 44, 5–10 participants per focus group) who were living in Minnesota (MN), between the ages of 30–65 years and eligible for cervical cancer screening. Participants were recruited through flyers at two partner clinics and two local community sites in urban centers in MN and word of mouth. Before the focus groups, participants completed a phone-based demographic survey which collected participant age, gender, country of birth, years living in the USA, race and ethnicity, primary language, household income, highest level of educational attainment, marital status, prior experience with cervical cancer screening, and connection to a primary care clinic. Five of the focus groups occurred in-person, and one was conducted over Zoom.

### Procedures

Focus groups were conducted in Somali by an experienced Somali American facilitator using a semi-structured guide developed by the Isbaar research team (See Table 1 for the guide). Focus group discussion areas covered women’s knowledge about HPV and cervical cancer, their screening experiences and views on HPV self-sampling, the influences on their decision making about cervical cancer screening, and their experience with clinic providers. The guide explored the interaction between how women think about HPV self-sampling, their behavior, and what socio-cultural factors impact their experience. Participants were asked how HPV self-sampling could best be implemented in primary care, including their perspectives on barriers and facilitators, informational needs, tailoring of instructions, and how it would best integrate into the clinic visit. To help inform the discussion, the focus group facilitator provided background information on HPV self-sampling and cervical cancer.

**Table 1:** Focus Group Discussion Guide Questions

Focus group discussion guide questions
Thank you for joining us for this discussion today. First, we will discuss some questions, then share some information with you all, and then we will continue our discussion. There are no right or wrong answers today. You might find that you agree or disagree with each other even, but we welcome all of your views and look forward to hearing all the different perspectives on this topic. As you know we are here to talk about cervical cancer screening today and we welcome all of your thoughts and experiences. Cervical cancer is a kind of cancer that starts in the cervix. Let’s take just a minute to review what the cervix is. As you might know, the uterus, or womb, is where babies develop during the course of a pregnancy. The cervix is the lower part of the uterus.
1. What has been your experience with cervical cancer screening, sometime described as the Pap test? Prompts: Have you had screening in the past? What was that experience like for you? What do you think about when you are deciding if you will have cervical cancer screening or not?
2. What has your experience with providers been like when having discussions about, or even having, cervical cancer screening?
3. When providers do cervical cancer screening, they are often checking for HPV, as it is a very common cause of cervical cancer. Have you heard about HPV testing before? Prompt: what are your thoughts about that?
Now we would like to describe HPV self-sampling to you. There is a new option for cervical cancer screening that may be available soon. This new option allows a woman to collect her own sample with a swab (like a long Q-tip). With self-sampling, a woman gently inserts the tip swab into her vagina and rotates for 10–30 s. She then removes the swab and places it in a vial or tube. The swab sample is then sent to a lab where it is tested for HPV. Her health care provider can then use the results from this HPV test to decide whether she needs additional tests to check for cervical cancer, or whether it is safe to wait several years before screening again.
4. What are your initial reactions to seeing this as a potential option for cervical cancer screening? Prompts: Is this something you would be interested in doing yourself? Why? Why not? What do you think about taking a sample yourself instead of the doctor taking the sample?
5. What would you need to know about HPV self-sampling to help decide if it was an option you were interested in or not?
6. If you had the chance to do this while at the clinic, how would you see that happening? Prompt: In the exam room? The sample bathroom? Who would you like to ask you about it?
7. In your view, what would be the best way to educate other Somali women about self-sampling for HPV?
Now we would like you to look at some instruction guides that woman could use to self-collect a sample.
8. What do you think about these instructions? Prompt, are they clear enough, would you still have questions about what to do? Would you like to have spoken or audio instructions in Somali?
10. What else would be helpful for the instructions to include? Prompt: How could we change these instructions to make them better?
11. Is there anything else we should know about these topics that would be helpful for serving the community and encouraging cervical cancer screening?
Thank you for your time today.

The groups lasted approximately an hour and were audio-recorded. All participants were provided with an information sheet about the study and verbally consented to participate; participants received a $50 gift card.

### Analysis

Focus data were translated and transcribed verbatim and analyzed with the assistance of NVivo software.^41^ Three study team members coded the transcripts systematically (HA, CBB, KF) and met regularly to review the emerging themes and subthemes. The analysis process was informed by the social constructivist approach to grounded theory, allowing for themes and sub-themes to emerge from the data while also considering the broader context, including theoretical frameworks and the literature.^42^ The broader group of research team members and the community advisory board further reviewed the emerging analysis to ensure the rigor of the analysis. Given the sample size and the many different perspectives within the Somali American community, we did not strive for data saturation or stratification of the data based on sub-groups; rather, we aimed to build a robust analysis. Inclusion of additional participants may have continued to contribute to our analysis or identified further potential barriers.

### Positionality

Our study team has both shared and different lived experiences and social identities from those of our study participants. Our investigator team includes one Somali American member, and we have a community advisory board comprised of both Somali American men and women (six members in total). Our different identities and experiences influence how we collect, analyze, and report data. Our team includes those with and those without lived experience of undergoing cervical cancer screening, and those who have and have not experienced interactions with healthcare that have been impacted by racial identity. We have reflected on the limitations our different identities may bring to the analysis process and tried to account for them. For example, our focus group facilitator identified as Somali American, all data was coded at least once in full by a Somali American research assistant, and we have shared emerging findings with the community advisory board and sought input throughout the analysis process from all team members.

### Human Subjects

The University of Minnesota Institutional Review Board provided ethical approval for the conduct of this study (STUDY00012408, SITE00001416). University of Washington IRB approval was granted through a reliance agreement with the University of Minnesota (STUDY00013359). All participants provided consent to take part in the focus groups. The study is registered with ClinicalTrials.gov (NCT05453006).

## FINDINGS

Here, we present the main themes and associated sub-themes, including the experience of cervical cancer screening, barriers to screening, views on HPV self-sampling, and strategies for increasing screening. Participant demographics can be found in Table 2, and additional illustrative quotes are in Table 3.

**Table 2:** Sociodemographic Characteristics of Focus Group Participants

Characteristics (self-report)	*n*	%
Total	44	100
Gender		
Female	44	100
Marital status		
Single	4	9
Married	29	66
Divorced	4	9
Widowed	3	7
Separated	4	9
Primary language		
Somali	42	95
No response	2	5
Race		
Black/African-American	44	100
Ethnicity		
Somali	44	100
Country of birth		
Somalia	44	100
Years living in the USA		
0–5 years	0	0
6–10 years	13	30
11–15 years	6	14
16–20 years	15	34
21–25 years	8	18
26–30 years	2	5
Years of formal education		
None	5	11
1–6 years	11	25
7–12 years	9	20
12 + years	19	43
Annual household income		
Less than $25,000	23	52
$25,000–$50,000	15	34
More than $50,000	5	11
Unknown	1	2
Prior Pap smear or cervical cancer screening		
Yes	33	75
No	11	25
Timeframe of prior Pap smear or screening		
Within the last 5 years	25	57
Between 6 and 10 years ago	8	18
No prior screening	11	25
Has primary care clinic		
Yes	39	89
No	5	11

**Table 3:** Additional Illustrative Quotes

Theme	Sub-theme	Illustrative quotes
Experience of cervical cancer screening	Have had screening in the past	“Likewise I did the last two years, since I came to this country I did screening 3 times, I did not have any problems, and every time my doctor was advising me to do the test. She was referring me to screenings including breast screening, and everything else and I didn’t meet any problems.” (Focus Group 6, Participant 3)
	Felt there wasn’t a choice or was screened but did not know why	“I was having a full checkup and they added this to it, I didn’t have a choice.” (Focus Group 6, Participant 1) “My doctor told me she is doing a Pap smear for me but she didn’t share anything about cancer screening. This was five years ago. My doctor did not tell me what the test is for, she only said she is using metal to see. She also called me recently to remind me that it has been 5 years and said you should make an appointment to go back for testing” (Focus Group 2, Participant 3)
	Pain, fear and circumcision	“For the Pap smear, when they are inserting a metal you feel pain, because your opening is small.” (Focus Group 1, Participant 9)
	Have not had screening	“I never did cervical cancer screening. No one asked me or told me anything about it and I did not ask for it.” (Focus Group 1, Participant 7)
	Suspicious and mistrust about screening	“There are a lot of women who really believe these metals cause disease and advise each other not to do it. Don’t use these metals, the metal is carrying the disease. If they insert you will be infected” (Focus Group 2, Participant 3)
	Aware of what screening entailed	“My doctor explained to me that HPV is the virus that causes cancer, it’s a virus that sits at the opening of the uterus, and if they don’t pick it with the biopsy it will spread and if it spreads it will be hard to treat. She explained to understand it very well” (Focus Group 2, Participant 9)
	Didn’t know about it	“I never did cervical cancer screening. No one asked me or told me anything about it and I did not ask for it” (Focus Group 1, Participant 7) “No doctor has ever talked to me about it!” (Focus Group 1, Participant 4)
Barriers to screening	Fear of cancer	“Some of the women don’t go to the doctor, fearing the doctor telling them they have some disease or condition. They said you shouldn’t go to the doctor, he will always say something is wrong and never without. A woman told me you should stop this doctor that you are going to all the time, because they will tell you bad news. I told her [it] is [a] good thing to be aware of your health condition” (Focus Group 5, Participant 1)
	Circumcision	“The second reason to refuse all the time is for circumcision, because of it women including those had children say they don’t want the metal instrument. The reason almost 97% are refusing is because of the circumcision.” (Focus Group 2, Participant 9)
	Hard to discuss with Somalis	“If you tell them, they say you are wishing it upon me.” (Focus Group 6, Participant 2)
	Modesty	“Also, they have faith in Allah and if not in severe pain, say “why should you uncover and expose my private parts to a non-Muslim?” (Focus Group 2, Participant 7)
	Positive views of screening	“However, I went through all these screenings and nothing happened to me, Thank God. I encourage people a lot too when they say why are they checking there (reproductive organ). I tell them if we are here, we have to do screening while healthy. It was said if the disease spreads nothing can be done about it. Likewise I do breast screening as well. Every year I have my appointment in July and I do it all the time.” (Focus Group 2, Participant 1)
Views on HPV Self -Sampling	Concerns about self-sampling	“I want the Pap smear and I don’t want this.” (Focus Group 1, Participant 3) “I trust the doctor to do the test for me.” (Focus Group 6, Participant 3) “How about if I insert something in the wrong place, hurt myself and bring about pain and illness.” (Focus Group 6, Participant)
	Prefer self-sampling	“It is better for me to take care of my test and bring it to the doctor instead of a man doing the screening and seeing my private parts. This is a shyness in us” (Focus Group 2, Participant 5) “Pap smear causes a lot of pain, but this one is easy, and will cause less pain.” (Focus Group 1, Participant 9) “Yes, that is a good idea. I would like that one. It's going to be used. People are running away from the other one because of the pain you know. So this one, I think this one people are going to use it. More Somalis are going to use it, because it is less painful, you know.” (Focus Group 4, Participant 3) “It's better in a few ways, first nobody is looking at you down there. The metal you were being inserted and pushed in down there is no more. This is a good idea.” (Focus Group 4)
	Feedback on materials or instructions	“It is just like the COVID test.” (Focus Group 1, Participant) “It’s easy.” (Focus Group 1, Participant 5) “The other one is covered the person has to read, but this one is clear. Some people may not be able to read. Do they insert it in the anus or in the front? The person has to understand, the person may make a mistake.” (Focus Group 4) “That person can ask the interpreter to explain to her. She will take out the guide and explain step by step showing pictures; you will open the tube, take out and insert to the red-mark, do you see the red-mark, twist it and then take it out without touching and put it back into the tube.” (Focus Group 1, Participant 10)
Strategies for increasing screening	Address fear and stigma:	“It’s believed that they don’t cure (save) Muslims. They would say we diagnosed you with cancer, over medicated you or burned you and they wouldn’t give you their best.” (Focus Group 3, Participant) “Some of the women don’t go to the doctor [for] fear from the doctor telling them they have some disease or condition. They said you shouldn’t go to the doctor, he will always say something is wrong and never without. A woman told me you should stop this doctor that you are going to all the time, because they will tell you bad news. I told her it is a good thing to be aware of your health condition.” (Focus Group 5, Participant 1)
	All clinics should offer self-sampling	“Everyone has a primary clinic. All clinics should be informed about this new system.” (Focus Group 1, Participant 2)
	Provider must encourage screening	“In my view it is better for the doctor to share with me what she doing to me; we are checking you for this, or this is what is happening, you are due for the test, it is good to be tested regularly at different times, so be aware of these things because if I am not told then I am not aware. I would like to understand what is being done, so when I come to meetings like these I can argue I have done the screening and was told this and that. But if she does not tell me anything about what she is doing then I won’t understand what she tested me for.” (Focus Group 5, Participant 4)
	Community awareness raising	“Today, each one of us came from home and the neighborhood, and [the] sister came to teach us something. You can take it back to your neighbors - this is a new tool, this is what they said, what do you think?” (Focus Group2, Participant 9) “Awareness at community locations, mosques where people gather. The people who study health, like the nurse women, women in health programs, to do more papers [brochures], even billboards, be played on TV, speaking in Somali, with the picture. A lot of awareness at where the Somalis gather.” (Focus Group 4)
	Individual encouragement and confidence	“Now, when you go to the doctor, if the doctor explains to you about the new test and shows you how to do it, you will know how to do it yourself and you will agree to the cancer screening. In my opinion if doctors educate people and raise their awareness, they will be heard and people will be encouraged” (Focus Group 6, Participant 3) “The way you share with us, we also share with others, and when someone says I did this more people will be encouraged and feel comfortable” (Focus Group 6, Participant 5)

### Experience of Cervical Cancer Screening

Participants had varying knowledge about and experiences of cervical cancer and/or cervical cancer screening. Of those who were aware of screening, some described having learned about cervical cancer screening through their provider. Some participants reported having had cervical cancer screening in the past, either as part of yearly checkups or related to a health concern. Many reported screenings to be a neutral or positive experience, and that the results they received were clear and understandable, although some expressed feeling initially afraid of the screening because they were unfamiliar with it or had concerns about the results. For some participants, cervical cancer screening has become a normal part of their care.

In contrast, among those who had been screened before, some reported having cervical cancer screening without understanding why it was being done, what the screening was for, how often it needed to be done, or that the provider did not explain the process thoroughly. A few participants shared that the provider did not ask for their permission before conducting the screening. This led to cervical cancer screening being described as a distressing, coercive, and frightening experience.

Yes, my doctor did a Pap smear test for me, looked inside, put fingers in me, and then sent me to another physician. I was feeling a bit of pain and they converged on me. They put things in me, inserted metal tools and sort. It was my first day, and like being in labor having a baby, I was scared that day and I was alone by myself. I didn’t know what they were doing and thought to myself " are they doing birth control to you?” and I was scared when I left. (Focus Group 3, Participant 6)

Other participants described having never been screened for cervical cancer. A number of respondents shared they had not been informed about HPV/cervical cancer or screening, or offered screening by their provider. Some participants shared a perception that providers do not discuss cancer with Somali people because of knowing that there is a fear of cancer in the community or because of a concern that patients will find it immodest.

### Barriers to Screening

Participants described a range of community-level barriers to undergoing cervical cancer screening, including a view that you only reach out to the doctor if you are sick or in pain. Because of this, participants emphasized that it is important for doctors to explain to patients that one can have cancer without feeling unwell. Some participants described that the community can be suspicious and mistrustful of cervical cancer screenings, including concerns that Western medical providers do not give their best care to Muslims, and that this is evidenced by the perception of there being few cancer survivors in the Somali community. Participants also described that people fear being ostracized and blamed within the Somali community for having a disease such as cancer, which may increase reluctance to screen. Some participants described a fear that by having screening, it is as if they are wishing disease upon themselves.

Participants shared concerns around the impact of female genital circumcision on undergoing cervical cancer screening. Having experienced circumcision was described as leading to greater concerns about modesty and pain. Many participants found it painful to have a Pap test due to being circumcised. Several participants also shared concerns about the use of the speculum in a physical exam. One belief discussed by participants was a concern that the speculum used is infected and causes cervical cancer. Even those who had given birth to children found that it was painful to have a speculum inserted into their vagina; one participant described the pain as similar to being in labor.

It was very painful. Too much click and clack of that metal. That metal is painful and I asked her ‘are you cutting a piece of flesh out’, she said ‘No, we just got a secretion specimen. I thought she was cutting part of my body.’ (Focus Group 5, Participant 3)

Some participants reported that faith and modesty were barriers to cervical cancer screening, particularly if the provider was male or non-Muslim. A few participants expressed a preference for female doctors, specifically Somali-speaking doctors, as it removed feelings of shyness, and generally, it was felt that a Somali person can be trusted. It was discussed that having a male provider could be acceptable if the person was in a lot of pain, but not acceptable for a routine screening.

I believe the main reason is culture and modesty, because most of the time doctors are male and every woman is refusing to show her private part to a man when there is no necessity or difficult circumstance (Focus Group 2, Participant 1)

### Views on HPV Self-Sampling

Due to varying levels of knowledge about cervical cancer screening, participants were provided an explanation of cervical cancer screening and of HPV self-sampling. The majority of participants had positive views of HPV self-sampling being offered in the clinic and trusted themselves to successfully perform the procedure. Some also felt it would be a good option to use at home. Advantages discussed included the ease and speed of the test, less pain, convenience, less invasive, and more privacy. Many believed HPV self-sampling would help overcome shyness and mistrust of doctors, making it more accessible and acceptable. Overall, participants widely support the self-sampling approach as a preferable and more comfortable alternative to prior screening methods.

Yes, that is a good idea. I would like that one. It’s going to be used. People are running away from the other one because of the pain you know. So, this one, I think this one people are going to use it. More Somalis are going to use it, because it is less painful you know (Focus Group 4, Participant 3)

Some participants had reservations about their ability to successfully collect a self-sampled test. Their concerns included the potential for discomfort, fear of incorrect insertion, and general uncertainty about the process, such as wondering about the provider’s role in a self-test and if Pap tests would still be necessary. One participant was mistrustful of the accuracy of the self-sampling results, citing reliability concerns with COVID-19 test results. A few felt their doctor could perform the screening more accurately and preferred the familiarity of the Pap test.

How about if I insert something in the wrong place, hurt myself and bring about pain and illness. (Focus Group 3)

When providing feedback on two versions of HPV self-sampling instructions, some felt that both versions were easy to understand, and participants expressed feeling familiarity with the test swab given its similarity to a COVID-19 test swab or an ear swab. Regarding images on the instructions, a few participants preferred the use of images that showed the patient wearing as much clothing as possible because they were more modest. Others preferred less clothed images because they felt they made the instructions more understandable. Participants discussed the importance of considering dialects when translating into Somali, and a number expressed concerns about a patient’s ability to read in English or Somali. They recommended additional modes of communication, including audio or video recordings. Others recommended that thorough instructions be given in-person by a nurse, interpreter, or community health worker.

### Strategies for Increasing Screening

Some participants stressed the importance of increasing self-confidence and knowledge, including through sharing of personal experiences, and encouraging individuals to engage in cervical cancer screening by offering HPV self-sampling. A few participants felt that some people in the Somali community lack confidence in health matters or do not always prioritize their own health, and that healthcare providers, interpreters, and other community members have a role in helping individuals feel more confident.

Participants emphasized the importance of diverse strategies to educate and raise awareness within the Somali community about cervical cancer and screenings/treatment. Information in the Somali community is often spread by word of mouth, and participants recommended informational group sessions at important community settings like mosques, clinics, community centers, and daycares/playgrounds as places to provide education about cervical cancer. Participants also recommended a variety of tools for relaying health information, including Somali television channels, educational brochures, videos, regular educational meetings, sermons at the mosque, text messages, billboards, and social media platforms (e.g., WhatsApp). It was mentioned that presentations by community members would be particularly effective, including community leaders, healthcare providers, and religious leaders.

You can educate them and raise their awareness by holding meetings like these, and then the Somali women will spread the word to other women. Everyone will talk to their friends and neighbors and show them how it’s done. (Focus Group 1, Participant 10)

Many participants expressed it was an individual’s responsibility to share what they have learned about health and screening with people they know, such as friends, neighbors, and co-workers, since close relationships are an effective way to spread information. By encouraging individuals to get screened, they felt it could have an impact on raising awareness and encouraging people to prioritize their health. A few people discussed the impact that everyday people could have as advocates for cervical cancer prevention. For example, it could be powerful to have individuals affected by cervical cancer or survivors share their stories to reduce the stigma associated with the disease.

So the amount of knowledge I have now is enough to go share with others at the daycare where I work and others will take it to their places. We’ll spread the word to the best of our abilities. (Focus Group 5, Participant 2)

Participants had many suggestions on how to increase and improve upon the experience of cervical cancer screening in clinics. In general, participants felt that both Pap and HPV self-sampling options for cervical cancer screening should be offered to patients with other preventative procedures at check-up visits. Some participants emphasized the importance of having the HPV self-sampling option available in all primary care clinics.

First the doctor must give you an explanation when you are there. She has to say we have two kinds of tests. You have two choices. This one like that… this this, she has to give you an explanation. After that to give the choice to the patient, the patient [can] choose which one they like. It’s possible that they will choose the previous design, and say I don’t want the new one, I know this one. So it depends on the person, the doctor or the nurse how she explains it to the patient. (Focus Group 4, Participant 1)

Participants also provided suggestions on provider education around cervical cancer screening. They looked to physicians for health guidance, and many felt that providers should be more proactive in educating on preventative health options; a few mentioned the importance of repeated reinforcement of the health information and asking the patient more than once during the visit about the screening. Participants relayed experiences with doctors who seemed rushed, provided incomplete information, and/or seemed unwilling to provide comprehensive information during appointments. Some participants mentioned feeling uncomfortable asking questions of the provider; participants also discussed the need to be proactive in asking questions about their health.

They don’t want to give you more time to give you information. I don’t know if they don’t have enough time [or] they have no time to explain things. They want to make everything short even if you want to ask questions and you want them to explain more. They don’t want you to keep talking, their time is calculated and measured, and they want you to leave the place (Focus Group 5, Participant 1)

The need for more or better-trained clinic interpreters was discussed by several participants. There is concern about inaccurate interpretation during visits and/or the interpreter being in a rush, which leads to frustration during health visits.

## DISCUSSION

HPV self-sampling is a promising strategy for individuals who may opt out of cervical screening^15^ and could help address cervical cancer screening disparities. Our findings indicate that HPV self-sampling may be a preferred option for some Somali American women, and at a minimum should be offered as an alternative to Pap and/or HPV testing on clinician-collected samples. Offering HPV self-sampling could be of vital importance to people who have had traumatic, coercive experiences, and for patients who have experienced female genital circumcision.

The literature on Somali women’s experiences in relation to cervical cancer screening illustrates concerns with limited health literacy,^25^ lack of knowledge about cancer,^26,27^ and limited awareness about HPV and cervical cancer.^26–30^ Our findings add to this literature by also exploring how limited knowledge and awareness can impact the experience of undergoing a Pap test. Some women expressed experiences in undergoing a Pap test that have been frightening, distressing, and at times coercive. While this was not the experience of all participants in this research, it was common, and signals that great care should be given in describing and conducting cervical cancer screening in this population in ways that are mindful that patients may have had a prior traumatic experience of screening. It also suggests there are opportunities to better educate and support providers to have greater awareness of the impact of prior screening experiences and to see HPV self-sampling as an important tool to support patient-centered screening.

Some participants were frustrated because they had never been screened and had never been offered cervical cancer screening by providers. This was perceived to be due to not wanting to cause offense or preconceived views about Somali American patients on the part of the provider. This contributes to the well-documented mistrust of healthcare providers and systems^25,29,36^ but also raises the challenge that providers and health systems themselves have an opportunity to act in ways that foster and build trust better. In relation to cervical cancer screening, this means consistently educating patients and offering screening, but doing so in ways that are cognizant of prior traumatic screening experiences. Providers may need more education and support, particularly if for some, not offering screening at all has become an alternative to navigating difficult conversations or topics. This may particularly be the case where providers are either non-Muslim or non-Somali, as mistrust can be higher where there is not patient-physician racial concordance^43–45^ or religious concordance.^46^

Our findings show that for Somali American women, HPV self-sampling was seen as a very good option, with few identified concerns. The concerns that were identified included concerns about the test’s reliability or worries about knowing how to collect the sample correctly. It was also seen as a very good option for patients who have concerns about modesty, which is a common view.^35^ A unique feature of this population is the high rate of female genital circumcision, which was identified as a prominent factor impacting the experience of screening, acting as a barrier to screening and intensifying modesty concerns.

Our findings support the potential of HPV self-sampling as a promising alternative for women who may opt out of cervical screening.^15^ However, our findings also suggest that for HPV self-sampling to reduce screening disparities, it will need to be offered with a thoughtful and comprehensive strategy that is inclusive of trauma-informed approaches and the need to make self-sampling readily available to those who may benefit the most.

Overall, our findings suggest that multilevel interventions and strategies may be needed to address cervical cancer screening within the Somali American community. These span the individual level, with a need to increase health literacy and engagement in preventive health. Another is the need to address provider and system level changes to support the needs of the community around cervical cancer screening. Finally, our findings also highlight the value of seeking community members’ input on how to address these persistent disparities, and that community engaged approaches in planning and deploying HPV self-sampling to address disparities will be key.

### Limitations

This qualitative study focused on urban settings in the upper Midwest in the USA. As such, it may not be generalizable to other settings or contexts. The participants in this study were recruited to share their views on cervical cancer screening and thus may have been predisposed to feeling open to discussing these topics. It should also be noted that the Somali American community is diverse, and these findings may not represent the views and experiences of all within the community.

## CONCLUSIONS

HPV self-sampling may be considered a preferred option for some patients, such as Somali American women, and at a minimum should be offered at the same time as Pap and/or HPV testing on clinician-collected samples when appropriate. Offering HPV self-sampling to Somali American women could be an important tool to address barriers related to modesty and access to care and may be most effective when implemented alongside education to raise awareness about cervical cancer. This modality may be particularly important for patient populations who have had traumatic or coercive screening experiences and for patients who have experienced female genital circumcision.

## Data Availability

The datasets used and/or analyzed during the current study are available from the corresponding author on reasonable request.

## Abbreviations

HPV: Human papillomavirus
WHO: World Health Organization
